# The impact of diabetes and osteoarthritis on the occurrence of stroke, acute myocardial infarction, and heart failure among older adults with non-valvular atrial fibrillation in Hawaii: a retrospective observational cohort study

**DOI:** 10.1186/s12889-021-11247-0

**Published:** 2021-06-21

**Authors:** Masako Matsunaga, John J. Chen, Mayumi Jijiwa, Eunjung Lim

**Affiliations:** Department of Quantitative Health Sciences, John A. Burns School of Medicine, University of Hawaii at Manoa, Honolulu, HI USA

**Keywords:** Non-valvular atrial fibrillation, Diabetes, Osteoarthritis, Cardiovascular disease, Stroke, Myocardial infarction, Heart failure, Older adults, Medicare

## Abstract

**Background:**

To date, little is known about cardiovascular disease risks among older adults with non-valvular atrial fibrillation by their association with diabetes and osteoarthritis status, based on longitudinal data with substantial amounts of non-white individuals. The objective of this study was to examine the risks for three cardiovascular diseases: stroke, acute myocardial infarction (AMI), and heart failure (HF), by diabetes and osteoarthritis status among older adults with non-valvular atrial fibrillation in Hawaii.

**Methods:**

We conducted a retrospective observational cohort study for older adults (65 years and older) with non-valvular atrial fibrillation using the Hawaii Medicare data 2009–2017. Their risks for the three cardiovascular diseases by diabetes and osteoarthritis status (diabetes, osteoarthritis, diabetes and osteoarthritis, and without diabetes and osteoarthritis) were examined by multivariable Cox proportional hazard regression models.

**Results:**

The analysis included 19,588 beneficiaries followed up for a maximum of 3288 days (diabetes: *n *= 4659, osteoarthritis: *n *= 1978, diabetes and osteoarthritis: *n *= 1230, without diabetes and osteoarthritis: *n *= 11,721).  Among them, those diagnosed with the cardiovascular diseases were identified (stroke: diabetes *n *= 837, osteoarthritis *n *= 315, diabetes and osteoarthritis *n *= 184, without diabetes and osteoarthritis *n *= 1630)(AMI: diabetes *n *= 438, osteoarthritis *n *= 128, diabetes and osteoarthritis *n *= 118, without diabetes and osteoarthritis *n *= 603)(HF: diabetes *n *= 2254, osteoarthritis *n *= 764, diabetes and osteoarthritis *n *= 581, without diabetes and osteoarthritis *n *= 4272). After adjusting for age, sex, race/ethnicity, and other potential confounders, those with diabetes and osteoarthritis had higher risks for HF (hazard ratio: 1.21 95% confidence interval: 1.10–1.33) than those without diabetes and osteoarthritis. They also had higher risks than those with osteoarthritis for HF. Those with diabetes had higher risks for all three cardiovascular diseases than the other three groups.

**Conclusions:**

Variation in cardiovascular disease risks for older adults with non-valvular atrial fibrillation in Hawaii exists with diabetes and osteoarthritis status.

**Supplementary Information:**

The online version contains supplementary material available at 10.1186/s12889-021-11247-0.

## Background

Stroke, acute myocardial infarction (AMI), and heart failure (HF) are among the leading causes of death in US older adults [1–4]. Individuals who survive these cardiovascular diseases (CVDs) often experience a low health-related quality of life (HRQOL) [5, 6]. In addition, it often results in increasing caregivers’ burden and health care expenses [7–9]. Although the CVD mortality and incidence rates decreased over recent years, health disparities in the rates still exist in multiple dimensions, including socioeconomic status, race/ethnicity, and health status [2, 4, 7].

Non-valvular atrial fibrillation (NVAF) is a common arrhythmia disease and a risk factor for CVDs, such as stroke and HF [4, 10, 11]. The number of patients with NVAF is projected to increase due to the growing older adult population [12]. Since older adults are more likely to have multiple chronic conditions, their CVD risks could depend on their comorbidity status. Furthermore, recovery from CVD is more challenging for individuals with other chronic diseases [13]. Thus, examining their CVD risks by other health conditions’ status may provide new insights on NVAF management. Such a study is necessary to develop public health strategies to decrease future healthcare crises. Also, an action plan is required for the community with a high proportion of members experiencing health disparities.

Risk factors for NVAF include metabolic disturbances [14, 15], such as diabetes mellitus (DM) and osteoarthritis (OA), which are also a health-status risk factor for CVD. OA, the most common form of arthritis, is the common cause of joint pain, physical disability, and low HRQOL among older adults [16]. Past studies reported poor lipid and glycemic profiles and a higher CVD incidence rate among those with OA than those without [17, 18]. Because adults with DM often develop OA and vice versa, recent studies examined the association between the two diseases [19, 20].

To date, little is known about CVD risks among older adults with NVAF by DM and OA status, especially among those in the community with a high proportion of non-white individuals. To fill this gap, we aimed to examine risks for stroke, AMI, and HF over time among older adults with NVAF by DM/OA status, using the Hawaii Medicare claims data 2009–2017. Because Hawaii’s population includes high proportions of Asians and Pacific Islanders (PI), our investigation is also vital to provide useful insights on developing future multifactorial prevention and intervention initiatives for those high CVD risk patients in a multiracial community. We hypothesized that older adults with NVAF who also have DM and OA are more likely to have higher risks for stroke, AMI, or HF than those without either DM or OA or both.

## Methods

### Data source and study population

We conducted a retrospective observational cohort study using 2009–2017 Hawaii Medicare inpatient, outpatient, and carrier claim files and beneficiary summary files. The University of Hawaii Human Studies Program approved this study. Using the International Classification of Diseases, Ninth or Tenth Revision, Clinical Modification (ICD-9-CM or ICD-10-CM), we identified individuals with NVAF, stroke, AMI, HF, DM, and OA from the claim files [21, 22] (Supplementary Table 1). The date on which a beneficiary was diagnosed with NVAF was treated as his or her baseline date.

Among the Medicare beneficiaries with Hawaii residency, 29,222 beneficiaries were identified as those with an NVAF diagnosis. Beneficiaries aged 64 years and younger (*n* = 2569) and beneficiaries who had a stroke, AMI, or HF diagnosis before their baseline date were excluded for analysis (*n* = 7065), resulting in 19,588 study subjects for the analysis. These individuals were classified into four mutually exclusive cohorts of DM/OA status: with both DM and OA (with DM/OA), with DM only (with DM), with OA only (with OA), or without DM and OA (without DM/OA), based on their diagnoses before the baseline date.

Those who had been diagnosed with each of the CVDs were categorized as “had a diagnosis” on their diagnosis dates, and days from the baseline date were calculated for each. Those who were not diagnosed with the CVD were censored on their last days in the study period, and days from their baseline date were calculated for each. If an individual’s DM/OA status changed during the study period, this individual was censored on the new diagnosis date. For example, an individual initially without OA was later diagnosed with OA after his/her baseline date, days from the baseline date to the OA diagnosis date were calculated, and s/he was censored on the OA diagnosis date. If an individual died of causes other than CVD, s/he was censored on the death date.

### Demographics and comorbidities

Age, gender, race, zip code, and dual eligibility for Medicare and Medicaid were obtained from the beneficiary summary files. Study subjects were categorized into three age groups based on age at baseline (65–74, 75–84, or ≥ 85 years). Their race/ethnicity categories (White, Asian, PI, Hispanic, and Other race) were determined by two Medicare race variables (Beneficiary Race Code and Research Triangle Institute Race Code). White, Hispanic, and Other Race individuals were identified according to the Beneficiary Race Code. Black and North American Native were included in Other Race due to their small population sizes in the study population (< 1% for both races). If both race codes indicated Asian and Asian/PI, this individual was categorized as an Asian. If the Beneficiary Race Code indicated other than Asian, but the Research Triangle Institute Race Code indicated Asian/PI, this individual was classified as a PI. Oahu is the most populous island in Hawaii with the most major hospitals. Thus the Oahu residency can serve as an indicator of resource availability as Hawaii has been facing physician shortages, especially on neighboring islands [23]. We identified the residency of Oahu for each subject based on their zip codes. Dual eligibility was defined as “yes” if a beneficiary received both Medicare and Medicaid at least for 12 consecutive months and used as a proxy for socioeconomic status. Hypertension and hyperlipemia are well-known factors for CVDs. The associations with CKD, COPD, and dementia were examined by past studies [7, 24–27]. We identified those with hypertension, hyperlipidemia, chronic kidney disease (CKD), chronic obstructive pulmonary disease (COPD), and dementia at the baseline from the claim files using appropriate ICD-9-CM or ICD-10-CM codes ( Supplementary Table 1).

### Statistical analysis

The characteristics of older adult beneficiaries with NVAF were summarized by DM/OA status. Differences in the characteristics across the DM/OA status were examined by chi-square tests for categorical variables and analysis of variance for continuous variables, respectively. Incidence rates of the three CVDs per 1000 person-years were computed, and differences between the DM/OA groups were compared. Times to the CVD diagnoses were illustrated using Kaplan-Meier curves. Log-rank tests with the Benjamini-Hochberg adjustment were used to compare diagnosis rates of the four DM/OA groups. Cox proportional hazard regression models were used to obtain crude and adjusted hazard ratios (HRs) and their 95% confidence intervals (CIs) of the CVD by DM/OA status. Covariates included in the adjusted model were age group, sex, race/ethnicity, Oahu residency, dual eligibility, hypertension, hyperlipidemia, CKD, COPD, and dementia. The assumption of the proportionality of hazards for each variable over time was evaluated using scaled Schoenfeld residuals and appropriate plots. Models for AMI and HF were stratified by CKD due to the unproportionate hazards between those with and without CKD. Multiple comparisons among the DM/OA status groups were tested using Tukey’s method. All analyses were conducted in R version 3.8 [28]. Statistical significance was assessed by a *p*-value of less than 0.05.

## Results

Table 1 shows the characteristics of the study population. More than 50% did not have DM and OA at baseline (without DM/OA). The second-largest group was those with DM, followed by those with OA. The smallest group had both DM and OA (with DM/OA), consisting of less than 7% of the study population. Those with DM were more likely to be younger, male, Asian or PI, and living in Oahu, and had a higher proportion of dual eligibility than the others, and had a higher proportion of hypertension, hyperlipidemia, COPD, dementia, and CKD than those without DM/OA. Those with OA were more likely to be older and female and had a higher proportion of whites than the others, and had higher proportions of hypertension, hyperlipidemia, COPD, dementia, and CKD than those without DM/OA. Those with DM/OA had greater proportions of hypertension, hyperlipidemia, CKD, and COPD than the others.

**Table 1:** Characteristics of older adults with non-valvular atrial fibrillation (NVAF)^a^ by diabetes mellitus (DM) and osteoarthritis (OA) status: Hawaii Medicare data 2009–2017

Characteristic	Without DM/OA *n* = 11,721 (59.8%)	With DM *n* = 4659 (23.8%)	With OA *n* = 1978 (10.1%)	With DM/OA *n* = 1230 (6.3%)
**Age in year, mean (SD)**				
	77.9 (8.76) ^b,c,d^	76.9 (8.24) ^c,d^	80.3 (8.38) ^d^	79.2 (7.78)
**Age group, n (%)**				
65-74y	4836 (41.3)^b,c,d^	2117 (45.4) ^c,d^	605 (30.6) ^d^	399 (32.4)
75-84y	4047 (34.5)	1650 (35.4)	742 (37.5)	509 (41.4)
≥ 85y	2838 (24.2)	892 (19.1)	631 (31.9)	322 (26.2)
**Gender, n (%)**				
Male	6577 (56.1) ^b,c,d^	2757 (59.2) ^c,d^	880 (44.5) ^d^	619 (50.3)
Female	5144 (43.9)	1902 (40.8)	1098 (55.5)	611 (49.7)
**Race/ethnicity, n (%)**				
White	4412 (37.6) ^b,c,d^	1010 (21.7) ^c,d^	893 (45.1) ^d^	333 (27.1)
Asian	3106 (26.5)	1449 (31.1)	499 (25.2)	371 (30.2)
Pacific Islander	2346 (20.0)	1213 (26.0)	329 (16.6)	299 (24.3)
Hispanic	519 (4.4)	296 (6.4)	84 (4.2)	72 (5.9)
Other	1338 (11.4)	691 (14.8)	173 (8.7)	155 (12.6)
**Residency, n (%)**				
Other Island	3960 (33.8) ^b^	1384 (29.7) ^c,d^	677 (34.2)	416 (33.8)
Oahu	7761 (66.2)	3275 (70.3)	1301 (65.8)	814 (66.2)
**Dual eligibility, n (%)**^**e**^				
	1677 (14.3) ^b,c^	787 (16.9) ^c,d^	242 (12.2)	157 (12.8)
**Hypertension, n (%)**				
	6814 (58.1) ^b,c,d^	3971 (85.2) ^c,d^	1623 (82.1) ^d^	1169 (95.0)
**Hyperlipidemia, n (%)**				
	5262 (44.9) ^b,c,d^	3507 (75.3) ^d^	1460 (73.8) ^d^	1144 (93.0)
**COPD, n (%)**	1278 (10.9) ^b,c,d^	588 (12.6) ^c,d^	400 (20.2) ^d^	292 (23.7)
**Dementia, n (%)**	1013 (8.6) ^b,c,d^	478 (10.3) ^c,d^	275 (13.9)	157 (12.8)
**CKD, n (%)**	1867 (15.9) ^b,c,d^	1837 (39.4) ^c,d^	475 (24.0)	570 (46.3)

^a^ Older adults with NVAF had no stroke, acute myocardial infarction, and heart failure at baseline (*n* = 19,588). ^b,c,d^ A significant difference between the corresponding group in the column and the ^b^DM, ^c^OA, or ^d^DM/OA group (*p *< 0.05). ^e^Dual eligible for Medicare and Medicaid for at least 12 consecutive months over the study period. *Abbreviations*: *SD* =  standard deviation; *CKD* =  chronic kidney disease; *COPD* =  chronic obstructive pulmonary disease

Table 2 shows the counts of events and event rates for stroke, AMI, and HF. Among the study population, 2966 stroke diagnoses (median: 580 follow-up days), 1287 AMI diagnoses (median: 677 follow-up days), and 7871 HF diagnoses (median: 308 follow-up days) were observed. The overall event rates for stroke, AMI, and HF were 60.5, 24.3, and 207.6 per 1000 person-years. The rates of the CVDs varied across the groups: ranging between 56.6–68.9 stroke diagnoses, 19.2–36.5 AMI diagnoses, and 184.8–269.6 HF diagnoses per 1000 person-years. Those with DM had a higher stroke rate (68.9/1000 person-years) than those without DM/OA (*p* < 0.001), and had a higher AMI rate (33.6/1000 person-years) and a higher HF rate (260.0/1000 person-years) than those without DM/OA and those with OA (*ps* < 0.001). Those with DM/OA had a higher AMI rate (36.5/1000 person-years) and a higher HF rate (269.6/person-years) than those without DM/OA (*ps* < 0.001) and those with OA (*p* < 0.01, *p* < 0.001).

**Table 2:** Event and event rates by the presence of osteoarthritis and/or diabetes among older adults with non-valvular atrial fibrillation^a^: Hawaii Medicare data 2009–2017

	Total	Without DM/OA *n* = 11,721	With DM *n* = 4659	With OA *n* = 1978	With DM/OA *n* = 1230
**Stroke**					
Diagnosis, n	2966	1630	837	315	184
Days, median^b^	580.0	534.0	653.0	656.5	692.5
Rate, per 1000 p-yr (95% CI)	60.5 (58.3–62.7)	56.6 (53.8–59.4)^c^	68.9 (64.3–73.7)	63.2 (56.4–70.5)	59.6 (51.3–68.9)
**Acute myocardial infarction**					
Diagnosis, n	1287	603	438	128	118
Days, median^b^	676.5	631.0	715.0	739.5	739.0
Rate, per 1000 p-yr (95% CI)	24.3 (23.0–25.6)	19.2 (17.7–20.8) ^c,d,e^	33.6 (30.5–36.9)^ d^	23.7 (19.8–28.2) ^e^	36.5 (30.2–43.7)
**Heart failure**					
Diagnosis, n	7871	4272	2254	764	581
Days, median^b^	308.0	305.0	289.0	366.5	331.5
Rate, per 1000 p-yr (95% CI)	207.6 (203.1–212.3)	184.8 (179.3–190.4) ^c,d,e^	260.0 (249.4–271.0) ^d^	192.4 (179.0–206.6) ^e^	269.6 (248.1–292.5)

^a^ Older adults with non-valvular atrial fibrillation had no stroke, acute myocardial infarction, and heart failure at baseline (*n* = 19,588). ^b^ Follow-up duration in days. ^c,d,e^ A significant difference between the corresponding group in the column and the ^c^DM, ^d^OA, or ^e^DM/OA group (*p* < 0.05). *Abbreviations*: per 1000 *p-yr* per 1000 person-years; *CI*  confidence interval

**Fig. 1:**
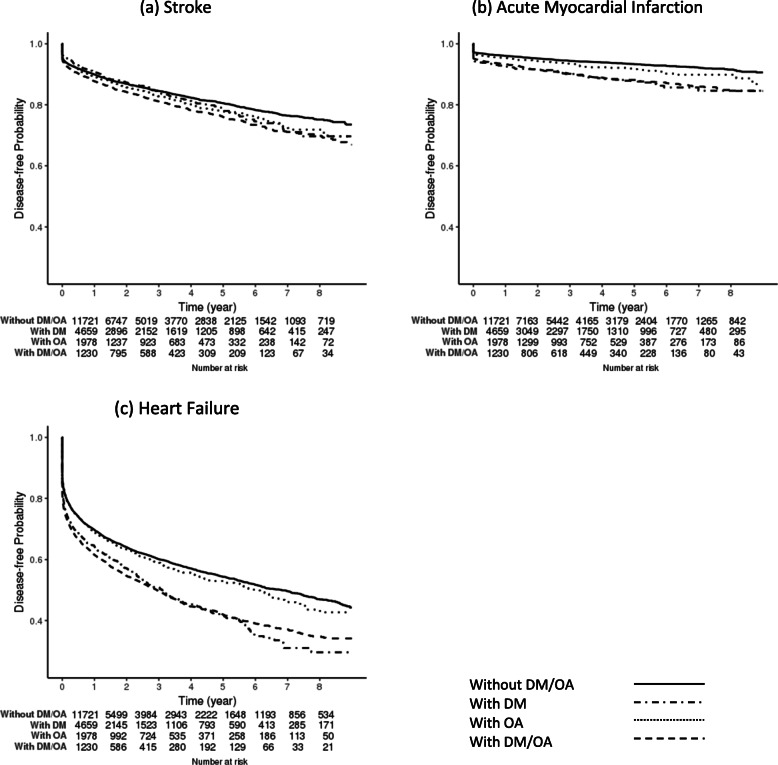
Kaplan-Meier curves for stroke, acute myocardial infarction, and heart failure among older adults with non-valvular atrial fibrillation by diabetes mellitus (DM) and osteoarthritis (OA) status: Hawaii Medicare data 2009–2017

Figure 1 illustrates Kaplan-Meier curves for (a) stroke, (b) AMI, and (c) HF. The time-to-first event curves show that the proportion of those developing HF was greater than those of developing stroke and AMI over the study period. Without DM/OA had the lowest probability of having any of the three CVD diagnosis, followed closely by with OA, and then with DM/OA and with DM. The disease-free probabilities for stroke were evenly spread among the four groups for stroke, and a significant difference was observed only between with DM and without DM/OA (pair-wise log-rank test: *p* < 0.001). However, this pattern was not found for AMI and HF: the curves for with DM and with DM/OA were closer to each other (pair-wise log-rank test: with DM vs. with DM/OA *p* > 0.05 for AMI and HF), and both were lower than without DM/OA (pair-wise log-rank test: with DM vs. without DM/OA *p* < 0.001; with DM/OA vs. without DM/OA *p* < 0.001 for AMI and HF), and with OA (pair-wise log-rank test: with DM vs. with OA *p* < 0.001 for AMI and HF; with DM/OA vs. with OA *p* = 0.002 for AMI, *p* < 0.001 for HF).

The results of Cox proportional hazard regression analysis (Supplementary Table 2) show that those with DM were 1.24 times more likely to be diagnosed with stroke (HR 1.24, 95% CI: 1.15–1.35), 1.80 times more likely to be diagnosed with AMI (HR 1.80, 95% CI: 1.59–2.04), and 1.38 times more likely to be diagnosed with HF (HR 1.38, 95% CI: 1.31–1.45) than those without DM/OA. Those with DM/OA were more likely to be diagnosed with AMI (HR 1.88, 95% CI: 1.54–2.29) and HF (HR 1.35, 95% CI: 1.23–1.47). The results of adjusted models (Table 3) show that the HRs of those with DM remained significant (stroke: 1. 29, 95% CI: 1.18–1.41; AMI: 1.31, 95% CI: 1.15–1.49; HF: 1.34, 95% CI: 1.26–1.41). Those with DM also had higher hazards than those with OA for all CVDs (stroke: *p* < 0.01, AMI: *p* = 0.02, HF: *p* < 0.001). Those with DM/OA were 1.21 times more likely to be diagnosed with HF (HR 1.21, 95% CI: 1.10–1.33) than those without DM/OA, but no difference was observed for stroke and AMI. Those with DM/OA also had higher risks than those with OA for HF (*p* < 0.001). No significant difference was observed in those with OA versus those without DM/OA for all the CVDs.

**Table 3:** Adjusted hazard ratios (HR) and 95% confidence intervals (CI) for stroke, acute myocardial infarction (AMI), and heart failure among older adults with non-valvular atrial fibrillation (NVAF)^a^: Hawaii Medicare data 2009–2017

	Stroke HR (95% CI) *P*^b^	AMI HR (95% CI) *P*^b^	Heart Failure HR (95% CI) *P*^b^
**Diabetes (DM)/osteoarthritis (OA) status (ref: without DM/OA)**			
With DM	1.29 (1.18–1.41) <.001	1.31 (1.15–1.49) <.001	1.34 (1.26–1.41) <.001
With OA	1.04 (0.92–1.18) 0.496	0.96 (0.79–1.17) 0.711	0.98 (0.90–1.06) 0.583
With DM/OA	1.02 (0.87–1.19) 0.830	1.15 (0.93–1.41) 0.193	1.21 (1.10–1.33) <.001
**Age group (ref: 65–74 years)**			
75–84 years	1.51 (1.39–1.65) <.001	1.40 (1.22–1.59) <.001	1.27 (1.21–1.34) <.001
≥85 years	1.84 (1.66–2.03) <.001	1.64 (1.40–1.92) <.001	1.64 (1.54–1.74) <.001
**Gender (ref: Male)**			
Female	1.12 (1.04–1.20) 0.004	0.85 (0.76–0.96) 0.006	0.94 (0.90–0.98) 0.006
**Race/ethnicity (ref: White)**			
Asian	1.10 (1.00–1.21) 0.054	1.12 (0.96–1.30) 0.145	1.05 (0.99–1.11) 0.132
Pacific Islander	1.04 (0.94–1.15) 0.471	1.02 (0.87–1.20) 0.801	0.95 (0.89–1.01) 0.102
Hispanic	1.09 (0.91–1.30) 0.364	1.43 (1.12–1.82) 0.004	1.05 (0.94–1.17) 0.374
Other	1.07 (0.95–1.22) 0.269	1.13 (0.93–1.36) 0.222	1.07 (0.99–1.15) 0.079
**Residency (ref: other island)**			
Oahu	1.10 (1.02–1.20) 0.019	0.99 (0.88–1.36) 0.935	0.88 (0.84–0.92) <.001
**Dual eligibility**^**c**^ **(ref: No)**			
Yes	0.91 (0.82–1.01) 0.086	0.87 (0.74–1.02) 0.089	1.08 (1.01–1.15) 0.017
**Hypertension (ref: No)**			
Yes	1.11 (1.02–1.22) 0.023	1.67 (1.42–1.98) <.001	1.14 (1.08–1.21) <.001
**Hyperlipidemia (ref: No)**			
Yes	0.92 (0.84–1.00) 0.042	1.29 (1.13–1.48) <.001	0.74 (0.71–0.78) <.001
**Chronic obstructive pulmonary disease (ref: No)**			
Yes	0.94 (0.84–1.06) 0.310	1.12 (0.96–1.31) 0.143	1.43 (1.35–1.52) <.001
**Dementia (ref: No)**			
Yes	1.28 (1.14–1.44) <.001	1.11 (0.93–1.33) 0.263	0.95 (0.88–1.03) 0.201
**Chronic Kidney Disease (ref: No)**			
Yes	0.91 (0.83–1.00) 0.053	NA	NA

^a^ Older adults with NVAF had no stroke, acute myocardial infarction, and heart failure at baseline (*n* = 19,588).^b^ *P*-values were obtained by testing the significance of regression coefficients in the Cox proportional hazards regression models.  ^c^ Dual eligible for Medicare and Medicaid for at least 12 consecutive months over the study period. *Note.* The model for stroke included DM/OA status, age group, gender, race/ethnicity, residency, dual eligibility, hypertension, hyperlipidemia, chronic obstructive pulmonary disease, dementia, and chronic kidney disease. The models for AMI and stroke included DM/OA status, age group, gender, race/ethnicity, residency, dual eligibility, hypertension, hyperlipidemia, chronic obstructive pulmonary disease, dementia, and were stratified by chronic kidney disease (No/Yes)

Older age and hypertension were associated with having a diagnosis for all three CVDs. Different patterns of the CVD hazard comparisons with the reference groups were found for sex (female: higher for stroke; lower for AMI and HF), race/ethnicity (Hispanic: higher for AMI), Oahu residency (higher for stroke; lower for HF), dual eligibility (higher for HF), hyperlipidemia (higher for AMI; lower for HF), COPD (higher for HF), and dementia (higher for stroke).

## Discussion

To the best of our knowledge, this is the first study that examined the hazard risks for stroke, AMI, and HF in the older adults with NVAF by DM/OA status in the community with a high proportion of non-White individuals, using Medicare data. We found that among adults with NVAF who aged 65 years and older in Hawaii, those with DM only had a higher diagnosis rate for stroke compared with those without DM/OA. For AMI and HF, those with DM/OA and those with DM only had similar diagnosis rates, which were higher than those with OA only and those without DM/OA. After adjusting for potential confounders, we observed the difference in hazards between those with and without DM/OA was significant for HF only. In contrast, the difference between those with DM only and without DM/OA remained significant for all three CVDs. Taken all together, these results indicate that having DM and OA together did not generate additive risks for any of the three CVDs to older adults with NVAF in Hawaii. Meanwhile, DM itself appeared to be a critical contributor to the risk for the three CVDs. However, those with DM in our study population were younger than the other groups. Younger individuals are more likely to be engaged with unhealthy behaviors, such as suboptimal dietary quality, excessive alcohol consumptions, and smoking [29, 30]. Although Medicare data allowed to examine the diagnosis and hazard ratios among US older adults, they did not include detailed sociodemographic data, such as income and  education. In addition, the CVD hazard risk differences by DM/OA status among older adults with NVAF may involve more complicated pathways than the general US older adult population. Further research with detailed sociodemographic and clinical data is needed to elucidate hazard differences across the DM/OA status among older adults with NVAF.

The current results showed that the overall diagnosis rate among older adults with NVAF was the highest for HF, followed by stroke and AMI. A possible reason for HF’s highest rate is that HF and NVAF are risk factors to each other [31, 32], and HF is a chronic and progressive disease and occurs more frequently than the other two CVDs in older adults [4]. Since NVAF is a well-known risk factor for stroke [4, 10, 11], it is not surprising that older adults with NVAF in the current study show the second-highest rate of stroke among the three CVDs. Of note, we found that the overall incidence rate of AMI among older adults with NVAF was the lowest. This may be because NVAF is not a direct risk factor for AMI. However, the results show that some older adults experienced such severe CVD. Since AMI could be fatal, a healthy lifestyle for AMI prevention should be included as a part of the CVD risk management for older adults with NVAF.

Numerous observational studies have reported the associations between OA and CVD risks. A recent meta-analysis study based on three longitudinal studies reported that patients with OA had higher risks for CVDs indicated by poorer atherosclerotic biomarkers and weight status and higher risk ratios for myocardial infarction and stroke, compared with patients without OA [18]. Another meta-analysis study based on 15 observational studies also reported that the risk for overall CVD was 1.24 times higher for patients with OA than the general population [17]. The study also reported high risks for ischemic heart disease, congestive heart failure but did not find a higher risk for stroke when the risks were stratified by types of CVDs. A different meta-analysis study based on 15 observational studies [33] found a higher prevalence of CVDs (38.4% vs. 9.0%) and higher relative risks for HF and ischemic heart disease among those with OA but not for myocardial infarction and stroke. These meta-analysis studies suggest that the degree of CVD risks in those with OA may depend on the type of the CVDs. The current study examined the CVD risks of OA patients by further categorizing them with DM status, which helps compare with the OA phenotypes suggested by past studies [34, 35]. The observed higher rate for CVDs in DM/OA group vs. OA only group could be related to the metabolic phenotype rather than other phenotypes associated with OA, such as the aging, inflammatory, genetic, and post-traumatic phenotypes. The current study did not take other important confounders into account. However, our findings may help elucidate the associations between OA phenotypes and CVD risks in future studies.

Our results show the associations of gender varied with the three CVDs. Female older adults with NVAF had a higher hazard for stroke but had a lower hazard for AMI and HF than mele counterparts. Previous studies reported that women had a higher risk for stroke [36, 37] and myocardial infarction [38], but a lower risk for HF [39]. However, a recent decreasing incidence rate of myocardial infarction among women has also been documented [38]. Although our study population was older and had more chronic diseases than the general adult population, the results appear consistent with the previous reports. We also observed various associations of the clinical conditions across the three CVDs. Those with hyperlipidemia had a higher hazard for stroke and AMI, but not for HF. Those with COPD had a higher hazard for HF, while those with dementia had a higher hazard for stroke. Hispanics had a higher hazard for AMI than White, which contradicted the previous report [2, 40]. Since Hispanics accounted for only 5% of the study population, our results may not be generalized to the US Hispanic population. However, the older Hispanic adults with NVAF in Hawaii appeared to have an additional burden for AMI. Another various hazard ratios across the CVDs were found for dual eligibility: beneficiaries eligible for Medicare and Medicaid had a higher risk for HF. The observed disparities could be related to the adherence level to the complex medical advice for NVAF management. For example, clinical cares for patients with NVAF often include oral anticoagulants to prevent blood clotting and manage blood flow to prevent further clinical complications [41]. However, suboptimal adherence to anticoagulants is common among patients with NVAF due to the necessity of routine monitoring for prothrombin time and special attention to avoid drug-drug and drug-food interactions [42]. Given that the prevalence of NVAF has been increasing due to growing numbers of older adults in the US, the characteristics of NVAF patients should be further examined to help develop better health care services, especially for minority and socially disadvantaged people.

The findings from the study should be interpreted with the consideration of several limitations. The severity of the CVDs and types of diabetes were not taken into account. Next, the CVD risks were assessed without adjusting for biomarkers and other potential risk factors (e.g., smoking, physical inactivity, obesity, family history, alcohol consumption, and dietary quality). Although diseases were identified from large datasets, including inpatient/outpatient claims (Medicare Part A and Part B) and carrier claims, claims from Advanced Medicare Plan Medicare (Medicare Part C) were not included in the current data. Thus, some misidentifications could exist. Comorbidity scores have been used for the prediction of stroke as well as other chronic diseases in recent studies [43, 44]. For example, the CHA2DS2-VASc score includes risk factors such as age, sex, prior stroke history, diabetes, and hypertension [45, 46]. Although the model also included other components for covariates, we might miss adjusting for all aspects from the comorbidity score. For those who changed their DM/OA status, their information after the time point of the new diagnosis was censored. Lastly, the results may not be generalized to older adults with NVAF in other regions with different race constitutions. Besides lifestyle/behavior factors, the frequencies of doctor visits, weight status, biomarkers, and medications were not included in this study.

Despite these limitations, the current study has several strengths. The use of a nine-year longitudinal dataset allowed more accurate CVD risk estimation compared with studies with cross-sectional data. Our data included a high proportion of non-White individuals, which also allowed the risk estimations for those minority groups that have been often overlooked in the past. Additionally, the use of Hawaii Medicare data enabled us to compare the variations in hazard ratios by DM/OA status based on all non-Health Maintenance Organization Medicare beneficiaries aged 65 years or older with NVAF in Hawaii. Finally, the finding that older diabetic patients with NVAF would have additional risks for the CVDs will provide a new and useful public health perspective. Healthcare professionals may need to consider developing multifactorial interventions for older adults with NVAF tailored to different DM/OA statuses.

## Conclusions

Among older adults, Medicare beneficiaries with NVAF in Hawaii, those with DM and OA had higher risks for HF than those with OA and without DM/OA, but did not have higher risks than those with DM. Although an additive effect of having DM and OA was not observed in the current study, the results suggest differences in the CVD risks exist with respect to DM/OA status. Further examination is required to identify clinical and pathological factors that contribute to the differences in CVD risks across DM/OA statuses.

## Supplementary Information

**Additional file 1: Supplementary Table 1.**. Definitions of variables.

**Additional file 2: Supplementary Table 2.** Crude hazard ratios and 95% confidence intervals for stroke, acute myocardial infarction, and heart failure among older adults with non-valvular atrial fibrillation by diabetes and osteoarthritis status: Hawaii Medicare data 2009–2017.

## Data Availability

The dataset supporting the conclusions of this article are available from the Research Data Assistance Center (ResDAC) from the Centers for Medicare and Medicaid Services. Restrictions apply to the availability of these data, which were used under license for the current study, and are not publicly available. However, data are available from authors upon reasonable request and with permission of ResDAC.

## Abbreviations

AMI: acute myocardial infarction
CI: confidence interval
CKD: chronic kidney disease
COPD: chronic obstructive pulmonary disease
CVD: cardiovascular disease
DM: diabetes mellitus
HF: heart failure
HRQOL: health-related quality of life
HR: hazard ratio
NVAF: non-valvular fibrillation
OA: osteoarthritis
PI: Pacific Islander
