# Supplementary material for: The impact of diabetes and osteoarthritis on the occurrence of stroke, acute myocardial infarction, and heart failure among older adults with non-valvular atrial fibrillation in Hawaii: a retrospective observational cohort study
Source: BMC Public Health. 2021 Jun 21;21:1183. doi: 10.1186/s12889-021-11247-0 (PMC8218453; doi:10.1186/s12889-021-11247-0)
Supplement: Supplementary file 1 — Additional file 1: Supplementary Table 1.. Definitions of variables. [file 12889_2021_11247_MOESM1_ESM.docx]

| Supplementary Table 1: Definitions of Variables | | | |
| --- | --- | --- | --- |
| Variable | Definition | ICD-9-CM | ICD-10-CM |
| Non-valvula atrial fibrillation (NVAF) | Those diagnosed with NVAF during the study period, identified from inpatient, outpatient, and carried claims by ICD-9-CM or ICD-10-CM [1] | 427.31  Exclude if a code for valvular heart disease was claimed (procedure code: 35.02, 35.12, 35.20, 35.22, 35.24, 35.26, 35.28; diagnosis code 394.0, 394.2, 396.1, 396.8, 396.9, 746.5, V43.3) | I48.0, I48.1, I48.11, I48.19, I48.2, I48.20, I48.21, I48.91  Exclude if a code for valvular heart disease was claimed (procedure code: 02QG, 02RF, 02RG, 02RH, 02RJ; diagnosis code: I05.0, I05.2, I08.0, I08.1, I08.3, I08.8, I08.9, I09.81, I34.2, Q23.2, Q23.8, Q23.9, Z95.2) |
| Diabetes (DM)^a^ | Yes or no to being diagnosed with DM, with OA, or with DM and OA, or without DM and OA before the NVAF diagnosis date (baseline date), identified from inpatient, outpatient, and carried claims by ICD-9-CM or ICD-10-CM [2] | 249, 249.01, 249.1, 249.11, 249.2, 249.21, 249.3, 249.31, 249.4, 249.41, 249.5, 249.51, 249.6, 249.61, 249.7, 249.71, 249.8, 249.81, 249.9, 249.91, 250, 250.01, 250.02, 250.03, 250.1, 250.11, 250.12, 250.13, 250.2, 250.21, 250.22, 250.23, 250.3, 250.31, 250.32, 250.33, 250.4, 250.41, 250.42, 250.43, 250.5, 250.51, 250.52, 250.53, 250.6, 250.61, 250.62, 250.63,  250.7, 250.71, 250.72, 250.73, 250.8, 250.81, 250.82, 250.83, 250.9, 250.91, 250.92, 357.2, 362.01, 362.02, 362.03, 362.04, 362.05, 362.06, 366.41 | E08.x, 00, E08.01, E08.10, E08.11, E08.21, E08.22, E08.3211, E08.3212, E08.3213, E08.3219, E08.329, E08.3291, E08.331, E08.3311, E08.3312, E08.3313, E08.3319, E08.339, E08.3399, E08.341, E08.3411, E08.3412, E08.3413, E08.3419, E08.3493, E08.3499, E08.351, E08.3511, E08.3512, E08.3513, E08.3523, E08.3529, E08.3531, E08.3532, E08.3533, E08.3539, E08.3549, E08.3551, E08.3552, E08.3553, E08.3559, E08.359, E08.3599, E08.36, E08.37X1, E08.37X2, E08.37X3, E08.37X9, E08.43, E08.44, E08.49, E08.51, E08.52, E08.59, E08.65, E08.622, E08.628, E08.630, E08.638, E08.641, E08.649, E09.29, E09.00, E09.01, E09.10, E09.11, E09.21, E09.22, E09.3292, E09.3211, E09.3212, E09.3213, E09.3219, E09.329, E09.3291,E09.3391, E09.331, E09.3311, E09.3312, E09.3313, E09.3319, E09.339, E09.349, E09.3399, E09.341, E09.3411, E09.3412, E09.3413, E09.3419, E09.3519, E09.3493, E09.3499, E09.351, E09.3511, E09.3512, E09.3513, E09.3541, E09.3523, E09.3529, E09.3531, E09.3532, E09.3533, E09.3539, E09.3591,  E09.3549, E09.3551, E09.3552, E09.3553, E09.3559, E09.359, E09.39, E09.3599, E09.36, E09.37X1, E09.37X2, E09.37X3, E09.37X9, E09.610, E09.43, E09.44, E09.49, E09.51, E09.52, E09.59, E09.65, E09.622, E09.628, E09.630, E09.638, E09.641, E09.649, E10.319,  E10.10, E10.11, E10.21, E10.22, E10.29, E10.311, E10.3299, E10.3213, E10.3219, E10.329, E10.3291, E10.3292, E10.3293, E10.3393, E10.3312, E10.3313, E10.3319, E10.339, E10.3391, E10.3392, E10.3492, E10.3411, E10.3412, E10.3413, E10.3419, E10.349, E10.3491, E10.36,  E10.351, E10.3511, E10.3512, E10.3513, E10.3519, E10.359, E10.43, E10.37X3, E10.37X9, E10.39, E10.40, E10.41, E10.42, E10.622, E10.52, E10.59, E10.610, E10.618, E10.620, E10.621, E11.00,E10.641, E10.649, E10.65, E10.69, E10.8, E10.9, E11.3212, E11.22, E11.29, E11.311, E11.319, E11.321, E11.3211, E11.3312,  E11.3291, E11.3292, E11.3293, E11.3299, E11.331, E11.3311, E11.3411, E11.339, E11.3391, E11.3392, E11.3393, E11.3399, E11.341, E11.351, E11.3419, E11.349, E11.3491, E11.3492, E11.3493, E11.3499, E11.3531, E11.3513, E11.3519, E11.3521, E11.3522, E11.3523, E11.3529, E11.3552, E11.3539, E11.3541, E11.3542, E11.3543, E11.3549, E11.3551, E11.37X1, E11.359, E11.3591, E11.3592, E11.3593, E11.3599, E11.36, E11.44,  E11.37X9, E11.39, E11.40, E11.41, E11.42, E11.43, E11.630, E11.610, E11.618, E11.620, E11.621, E11.622, E11.628, E13.10,E11.65, E11.69, E11.8, E11.9, E13.00, E13.01, E13.3219, E13.311, E13.319, E13.321, E13.3211, E13.3212, E13.3213, E13.3319, E13.3293, E13.3299, E13.331, E13.3311, E13.3312, E13.3313, E13.3419, E13.3393, E13.3399, E13.341, E13.3411, E13.3412, E13.3413, E13.3519, E13.3493, E13.3499, E13.351, E13.3511, E13.3512, E13.3513, E13.3542, E13.3529, E13.3531, E13.3532, E13.3533, E13.3539, E13.3541, E13.40, E13.3552, E13.3553, E13.3559, E13.359, E13.36, E13.39, E13.620, E13.49, E13.51, E13.52, E13.59, E13.610, E13.618, E13.9, E13.638, E13.641, E13.649, E13.65, E13.69, E13.8. |
| Osteoarthritis (OA)^a^ | Yes or no to being diagnosed with OA before the NVAF diagnosis date (baseline date), identified from inpatient, outpatient, and carried claims by ICD-9-CM or ICD-10-CM [2] | 715.00, 715.04, 715.09, 715.10, 715.11, 715.12, 715.13, 715.14, 715.15, 715.16, 715.17, 715.18, 715.20, 715.21, 715.22, 715.23, 715.24, 715.25, 715.26, 715.27, 715.28, 715.30, 715.31, 715.32, 715.33, 715.34, 715.35, 715.36, 715.37, 715.38, 715.80, 715.89, 715.90, 715.91, 715.92, 715.93, 715.94, 715.95, 715.96, 715.97, 715.98, 720.0, 721.0, 721.1, 721.2, 721.3, 721.90, 721.91 | M15.0, M15.1, M15.2, M15.3, M15.4, M15.8, M15.9, M16.0, M16.10, M16.11, M16.12, M16.2, M16.30, M16.31, M16.32, M16.4, M16.50, M16.51, M16.52, M16.6, M16.7, M16.9, M17.0, M17.10, M17.11, M17.12, M17.2, M17.30, M17.31, M17.32, M17.4, M17.5, M17.9, M18.0, M18.10, M18.11, M18.12, M18.2, M18.30, M18.31, M18.32, M18.4, M18.50, M18.51, M18.52, M18.9, M19.011, M19.012, M19.019, M19.021, M19.022, M19.029, M19.031, M19.032, M19.039, M19.041, M19.042, M19.049, M19.071, M19.072, M19.079, M19.111, M19.112, M19.119, M19.121, M19.122, M19.129, M19.131, M19.132, M19.139, M19.141, M19.142, M19.149, M19.171, M19.172, M19.179, M19.211, M19.212, M19.219, M19.221, M19.222, M19.229, M19.231, M19.232, M19.239, M19.241, M19.242, M19.249, M19.271, M19.272, M19.279, M19.90, M19.91, M19.92, M19.93 |
| Stroke | Yes or no to being diagnosed with stroke after the NVAF diagnosis date (baseline date), identified from inpatient, outpatient, and carried claims by ICD-9-CM or ICD-10-CM [1] | 433.01, 433.1, 433.11, 433.21, 433.31, 433.81, 433.91, 434.00, 434.01, 434.11, 434.91, 435.0, 435.1, 435.3, 435.8, 435.9, 436 | G93.49, G45.0, G45.1, G45.2, G45.8, G45.9, I63.00, I63.02, I63.011, I63.012, I63.013, I63.019, I63.02, I63.031, I63.032, I63.039, I63.09, I63.10, I63.111, I63.112, I63.119, I63.12, I63.131, I63.132, I63.139, I63.19, I63.20, I63.211, I63.212,I63.213, I63.219, I63.22, I63.231, I63.232, I63.233, I63.239, I63.29, I63.30, I63.311, I63.312,I63.313, I63.319, I63.321, I63.322, I63.323, I63.329, I63.331, I63.332, I63.333, I63.339, I63.341, I63.342, I63.343, I63.349, I63.39, I63.40, I63.411, I63.412, I63.413, I63.419  I63.421, I63.422, I63.423, I63.429, I63.431, I63.432, I63.433, I63.439, I63.441, I63.442, I63.443, I63.449  I63.49, I63.50, I63.511, I63.512, I63.513, I63.519, I63.521, I63.522, I63.523, I63.529, I63.531, I63.532, I63.533, I63.539, I63.541, I63.542, I63.543, I63.549, I63.59, I63.6, I63.8, I63.9, I67.89, I74, I74.0, I74.01, I74.09, I74.1, I74.10, I74.11, I74.19, I74.2, I74.3, I74.4, I74.5, I74.8 |
| Acute myocardial infarction (AMI) | Yes or no to being diagnosed with AMI after the NVAF diagnosis date (baseline date), identified from inpatient, outpatient, and carried claims by ICD-9-CM or ICD-10-CM [2] | 410.01, 410.11, 410.21, 410.31, 410.41, 410.51, 410.61, 410.71, 410.81, 410.91 | I21.01, I21.02, I21.09, I21.11, I21.19, I21.21, I21.29, I21.3, I21.4, I21.9, I21.A1, I21.A9, I22.0, I22.1, I22.2, I22.8, I22.9 |
| Heart failure (HF) | Yes or no to being diagnosed with HF after the NVAF diagnosis date (baseline date), identified from inpatient, outpatient, and carried claims by ICD-9-CM or ICD-10-CM [2] | 398.91, 402.01, 402.11, 402.91, 404.01, 404.03, 404.11, 404.13, 404.91, 404.93, 428.0, 428.1, 428.20, 428.21, 428.22, 428.23, 428.30, 428.31, 428.32, 428.33, 428.40, 428.41, 428.42, 428.43, 428.9 | I09.81, I11.0, I13.0, I13.2, I50.1, I50.20, I50.21, I50.22, I50.23, I50.30, I50.31, I50.32, I50.33, I50.40, I50.41, I50.42, I50.43, I50.810, I50.811, I50.812, I50.813, I50.814, I50.82, I50.83, I50.84, I50.89, I50.9 |
| Hypertension | Yes or no to being diagnosed with hypertension before the NVAF diagnosis date (baseline date), identified from inpatient, outpatient, and carried claims by ICD-9-CM or ICD-10-CM [2] | 362.11, 401.0, 401.1, 401.9, 402.00, 402.01, 402.10, 402.11, 402.90, 402.91, 403.00, 403.01, 403.10, 403.11, 403.90, 403.91, 404.00, 404.01, 404.02, 404.03, 404.10, 404.11, 404.12, 404.13, 404.90, 404.91, 404.92, 404.93, 405.01, 405.09, 405.11, 405.19, 405.91, 405.99, 437.2 | H35.031, H35.032, H35.033, H35.039, I10, I11.0, I11.9, I12.0, I12.9, I13.0, I13.10, I13.11, I13.2, I15.0, I15.1, I15.2, I15.8, I15.9, I67.4, N26.2 |
| Hyperlipidemia | Yes or no to being diagnosed with hyperlipidemia before the NVAF diagnosis date (baseline date), identified from inpatient, outpatient, and carried claims by ICD-9-CM or ICD-10-CM [2] | 272.0, 272.1, 272.2, 272.3, 272.4 | E78.0, E78.00, E78.01, E78.1, E78.2, E78.3, E78.4, E78.41, E78.49, E78.5 |
| Chronic kidney disease (CKD) | Yes or no to being diagnosed with CKD before the NVAF diagnosis date (baseline date), identified from inpatient, outpatient, and carried claims by ICD-9-CM or ICD-10-CM [2] | 016.00, 016.01, 016.02, 016.03, 016.04, 016.05, 016.06, 095.4, 189.0, 189.9, 223.0, 236.91, 249.40, 249.41, 250.40, 250.41, 250.42, 250.43, 271.4, 274.10, 283.11, 403.01, 403.11, 403.91, 404.02, 404.03, 404.12, 404.13, 404.92, 404.93, 440.1, 442.1, 572.4, 580.0, 580.4, 580.81, 580.89, 580.9, 581.0, 581.1, 581.2, 581.3, 581.81, 581.89, 581.9, 582.0, 582.1, 582.2, 582.4, 582.81, 582.89, 582.9, 583.0, 583.1, 583.2, 583.4, 583.6, 583.7, 583.81, 583.89, 583.9, 584.5, 584.6, 584.7, 584.8, 584.9, 585.1, 585.2, 585.3, 585.4, 585.5, 585.6, 585.9, 586, 587, 588.0, 588.1, 588.81, 588.89, 588.9, 591, 753.12, 753.13, 753.14, 753.15, 753.16, 753.17, 753.19, 753.20, 753.21, 753.22, 753.23, 753.29, 794.4 | A18.11, A52.75, B52.0, C64.1, C64.2, C64.9, C68.9, D30.00, D30.01, D30.02, D41.00, D41.01, D41.02, D41.10, D41.11, D41.12, D41.20, D41.21, D41.22, D59.3, E08.21, E08.22, E08.29, E08.65, E09.21, E09.22, E09.29, E10.21, E10.22, E10.29, E10.65, E11.21, E11.22, E11.29, E11.65, E13.21, E13.22, E13.29, E74.8, I12.0, I12.9, I13.0, I13.10, I13.11, I13.2, I70.1, I72.2, K76.7, M10.30, M10.311, M10.312, M10.319, M10.321, M10.322, M10.329, M10.331, M10.332, M10.339, M10.341, M10.342, M10.349, M10.351, M10.352, M10.359, M10.361, M10.362, M10.369, M10.371, M10.372, M10.379, M10.38, M10.39, M32.14, M32.15, M35.04, N00.0, N00.1, N00.2, N00.3, N00.4, N00.5, N00.6, N00.7, N00.8, N00.9, N01.0, N01.1, N01.2, N01.3, N01.4, N01.5, N01.6, N01.7, N01.8, N01.9, N02.0, N02.1, N02.2, N02.3, N02.4, N02.5, N02.6, N02.7, N02.8, N02.9, N03.0, N03.1, N03.2, N03.3, N03.4, N03.5, N03.6, N03.7, N03.8, N03.9, N04.0, N04.1, N04.2, N04.3, N04.4, N04.5, N04.6, N04.7, N04.8, N04.9, N05.0, N05.1, N05.2, N05.3, N05.4, N05.5, N05.6, N05.7, N05.8, N05.9, N06.0, N06.1, N06.2, N06.3, N06.4, N06.5, N06.6, N06.7, N06.8, N06.9, N07.0, N07.1, N07.2, N07.3, N07.4, N07.5, N07.6, N07.7, N07.8, N07.9, N08, N13.1, N13.2, N13.30, N13.39, N14.0, N14.1, N14.2, N14.3, N14.4, N15.0, N15.8, N15.9, N16, N17.0, N17.1, N17.2, N17.8, N17.9, N18.1, N18.2, N18.3, N18.4, N18.5, N18.6, N18.9, N19, N25.0, N25.1, N25.81, N25.89, N25.9, N26.1, N26.9, Q61.02, Q61.11, Q61.19, Q61.2, Q61.3, Q61.4, Q61.5, Q61.8, Q62.0, Q62.2, Q62.10, Q62.11, Q62.12, Q62.31, Q62.32, Q62.39, R94.4 |
| Chronic obstructive pulmonary disease (COPD) | Yes or no to being diagnosed with COPD before the NVAF diagnosis date (baseline date), identified from inpatient, outpatient, and carried claims by ICD-9-CM or ICD-10-CM [2] | 490, 491.0, 491.1, 491.8, 491.9, 492.0, 492.8, 491.20, 491.21, 491.22, 494.0, 494.1, 496 | J40, J41.0, J41.1, J41.8, J42, J43.0, J43.1, J43.2, J43.8, J43.9, J44.0, J44.1, J44.9, J47.0, J47.1, J47.9 |
| Dementia | Yes or no to being diagnosed with dementia before the NVAF diagnosis date (baseline date), identified from inpatient, outpatient, and carried claims by ICD-9-CM or ICD-10-CM [3] | 290.0, 290.1, 290.10, 290.11, 290.12, 290.13, 290.2, 290.20, 290.21, 290.3,290.4, 290.40, 290.41, 290.42, 290.43, 294.0, 294.1, 294.10, 294.11, 294.2, 294.20,294.21, 294.8, 331.0, 331.1, 331.11, 331.19, 331.2, 331.7, 331.82, 797 | F03.90, F01.50, F01.51, F04, F02.80, F02.81, F03.90, F03.91, F06.1, F06.8,G30.0, G30.1, G30.8, G30.9, G31.01, G31.09, G31.0, G31.1, G94, G31.83, R41.81 |
| ^a^ Used to make the DM/OA status variable (with DM, with OA, with DM and OA, without DM and OA). | | | |

[1] Rose AJ, Goldberg R, McManus DD, Kapoor A, Wang V, Liu W, et al. Anticoagulant Prescribing for Non-Valvular Atrial Fibrillation in the Veterans Health Administration. J Am Heart Assoc. 2019;8:e012646. <https://doi.org/10.1161/JAHA.119.012646>

[2] Chronic Conditions Data Warehouse. Condition Categories. <https://www2.ccwdata.org/web/guest/condition-categories>; 2020 [accessed April, 16 2021].

[3] Maust DT, Strominger J, Kim HM, Langa KM, Bynum JPW, Chang CH, et al. Prevalence of Central Nervous System-Active Polypharmacy Among Older Adults With Dementia in the US. JAMA. 2021;325:952-61. <https://doi.org/10.1001/jama.2021.1195>
