# Supplementary material for: The impact of diabetes and osteoarthritis on the occurrence of stroke, acute myocardial infarction, and heart failure among older adults with non-valvular atrial fibrillation in Hawaii: a retrospective observational cohort study
Source: BMC Public Health. 2021 Jun 21;21:1183. doi: 10.1186/s12889-021-11247-0 (PMC8218453; doi:10.1186/s12889-021-11247-0)
Supplement: Supplementary file 2 — Additional file 2: Supplementary Table 2. Crude hazard ratios and 95% confidence intervals for stroke, acute myocardial infarction, and heart failure among older adults with non-valvular atrial fibrillation by diabetes and osteoarthritis status: Hawaii Medicare data 2009–2017. [file 12889_2021_11247_MOESM2_ESM.docx]

| **Supplementary Table 2** Crude hazard ratios (HR) and 95% confidence intervals (CI) for stroke, acute myocardial infarction, and heart failure among older adults with non-valvular atrial fibrillation by diabetes (DM) and osteoarthritis (OA) status^a^: Hawaii Medicare data 2009-2017. | | | | | |
| --- | --- | --- | --- | --- | --- |
|  | **Stroke**  **HR (95% CI)** | **Acute Myocardial Infarction**  **HR (95% CI)** | | | **Heart Failure**  **HR (95% CI)** |
| Without DM/OA  With DM  With OA  With DM/OA | Reference  1.24 (1.15-1.35)***  1.12 (0.99-1.26)  1.04 (0.89-1.21) | | Reference  1.80 (1.59-2.04)***  1.24 (1.03-1.50)**  1.88 (1.54-2.29)*** | Reference  1.38 (1.31-1.45)***  1.04 (0.96-1.12)  1.35 (1.23-1.47)*** | |
| ^a^ Older adults with non-valvular atrial fibrillation who had no stroke, acute myocardial infarction, and heart failure at baseline (n=19,588). * *p*<.05, ** *p*<.01, *** *p*<.001 *Note.* *P*-values indicate a statistically significant difference from the reference group (H0: the difference is zero). | | | | | |
